# A typology of North Sea oil and gas platforms

**DOI:** 10.1038/s41598-022-11975-2

**Published:** 2022-05-16

**Authors:** J. M. Lawrence, P. G. Fernandes

**Affiliations:** School of Biological Sciences, University of Aberdeen, Aberdeen, AB24 2TZ UK

**Keywords:** Environmental sciences, Environmental impact, Ocean sciences

## Abstract

Since the commercial exploitation of marine oil and gas reserves began in the middle of the twentieth century, extensive networks of offshore infrastructure have been installed globally. Many of the structures are now nearing the end of their operational lives and will soon require decommissioning, generating renewed interest in their environmental impacts and in the ecological consequences of their removal. However, such work requires selection of a subsample of assets for surveying; censuses of the entire ‘population’ in any given jurisdiction are practically impossible due to their sheer number. It is important, therefore, that the selected sample is sufficiently representative of the population to draw generalized conclusions. Here, a formal clustering methodology, partitioning around medoids, was used to produce a typology of surface-piercing oil and gas platforms in the North Sea. The variables used for clustering were hydrocarbon product, operational state, platform design and material, and substructure weight. Assessing intra-cluster variability identified 13 clusters as the optimum number. The most important distinguishing variable was platform type, isolating floating platforms first, then concrete gravity-based and then fixed steel. Following clustering, a geographic trend was evident, with oil production more prevalent in the north and gas in the south. The typology allows a representative subset of North Sea oil and gas platforms to be selected when designing a survey, or an assessment of the representativeness of a previously selected subset of platforms. This will facilitate the efficient use of the limited funding available for such studies.

## Introduction

Many man-made structures have been installed throughout the world’s oceans, where they inevitably interact with the environment in which they are situated. These structures affect many aspects of the physics and biology of the ecosystem in which they are placed, e.g. altering water flow^1–3^ and providing hard substrate on which sessile organisms can settle and grow^4–9^. The complex 3D structure can also act as a nursery ground^10,11^ and attract more mobile species from the surrounding area^12–15^. As such they can provide essential habitat, leading to areas of elevated local biodiversity^16^ and animal density^14,17–19^. This in turn can result in increased feeding opportunities for predators at higher trophic levels^9,20–22^. The interactions between a structure and its environment will vary with many aspects of the structure’s physical properties, including the material from which it is constructed^4,23^, its size, and shape^24^. They may also be affected by the properties of the location in which it is situated, included water movement patterns, water depth, and the availability of similar alternative natural (or unnatural) structures or substrates^25^.

Due to both the proximity to shore and the relative shallow depth of the seabed, the majority of human activity in the oceans is concentrated on the continental shelves. These areas are also where most man-made structures have been installed^26–28^, in particular in those accessible areas with reserves of valuable natural mineral resources. The northern Gulf of Mexico, the Persian Gulf, and the North Sea, for example, contain very high densities of man-made structures, installed as part of a network of offshore oil and gas infrastructure. These structures show extreme diversity in their size, form and function, including pipelines, subsea manifolds, wellheads and templates, and surface piercing platforms. Even within these broad classes of infrastructure type, there can be a great variety in the size and form of these structures; for example, pipelines can be exposed, buried, or covered, and can form spans. Other subsea infrastructure such as manifolds and anchor moorings can range greatly in their size and complexity. However, it is the surface piercing platforms which perhaps show the greatest diversity in size and design. The platforms themselves can be fixed (drilled into the seabed), floating (and kept in place by a system of mooring lines), or gravity-based (resting on the seabed, held in place simply by the weight of the structure itself). They can be made from either concrete or steel. Even platforms of the same style and material can be very different, with, for example, fixed steel platforms ranging from small normally-unmanned monopod platforms to large complexes consisting of several large, bridge-linked, 4- 6- or 8-legged platforms.

In the North Sea, as elsewhere, much of the current oil and gas infrastructure has been in place for several decades. It is now at, or nearing the end of, its operational life, such that it will soon require decommissioning^29,30^. The North Sea is currently covered by legislation which states that (with some exceptions) ‘the dumping, and leaving wholly or partly in place, of disused offshore installations with the maritime area is prohibited’^31,32^, and so decommissioning will involve the complete removal of these installations and associated infrastructure. As well as the large financial costs associated with the break-down and removal of these structures, there will also be potentially significant environmental impacts of the decommissioning process, through seabed disturbance^33,34^, potential contamination risk^33,35,36^, and indeed, through the removal of the habitat and opportunities afforded to the local flora and fauna by the structures’ physical presence^37^.

This has led to renewed interest in studying the ecological and environmental impacts of these structures. For regulators to make informed decisions about decommissioning options, more information is needed about the roles played by these structures within the North Sea ecosystem, and the potential impacts of their decommissioning and removal. To this end, many environmental studies have looked to describe or investigate the biology and ecology of these systems, with many more currently underway (e.g. INSITE, www.insitenorthsea.org). However, one thing that is rarely considered, or can be limiting to the broad utility of a study’s findings, is the selection of a representative sample of structures at which to collect data.

With such a large number of offshore oil and gas assets in the North Sea, it is practically (and, normally, financially) impossible to conduct in-depth sampling at all locations, e.g. sampling at every single surface piercing platform throughout the region. The environmental interactions and ecological impacts of two different structures may be vastly different, however, due to differences in their physical shape, size and design. As such, it may be impossible to extrapolate the findings of a study conducted on only a small number of a single type of platform, to other platform types and locations, and so the conclusions may not be useful when considering ecosystem-wide management planning.

To enhance the applicability of the findings of such studies, ensuring efficient use of the limited funding available (by eliminating the need to repeat the work for a different type of structure), it is essential that a representative sample of structures is selected. Alternatively, if a subsample has already been selected and surveyed, it is important to understand how representative the subsample is of the wider population, so that any limitations of the conclusions can be acknowledged.

To a) select a representative subsample before data collection or b) assess the representativeness of a previously selected subsample from the population of North Sea oil and gas platforms, a formal typology is required, whereby platforms are classified into clusters based on common characteristics. This will mean that, based on the relevant variables selected on which to base the clustering, variability within clusters is much lower than variability between clusters. The relative split of the population between clusters can then be used to either select a representative sample, or to assess the representativeness of a previously selected sample.

## Methods

To create a formal typology, a comprehensive list of the items to be clustered [platforms in this case] is required, along with the corresponding complete dataset of variables on which the clustering will be based. Here, the OSPAR inventory of offshore installations^38^ was used for both the list of the ‘population’ of offshore platforms (n = 552) and the variables of interest to be used for the clustering. The variables selected were: hydrocarbon product, platform type, operational status, and substructure weight. Other variables were considered (e.g. water depth, whether the platform is manned or unmanned, latitude and longitude, and produced water disposal method), but were not included for reasons given later, see “Discussion”).

These variables include both categorical and continuous data, and so it was necessary to select a clustering methodology that performs effectively with mixed datasets. Partitioning around medoids^39^ (PAM) has previously been used for clustering with mixed categorical and continuous data, for a wide variety of applications, including, for example, identifying the psychological effects of COVID-19^40^, clustering fishing vessels into discrete fleets^41^, grouping Indonesian districts for priority for intervention to address stunting^42^, grouping estuaries by a range of biotic and abiotic factors^43^, grouping similar patients presenting with back pain^44^, and identifying different fishing tactics from catch composition^45^ among others^46–48^.

Prior to the execution of a clustering algorithm, some measure of the distance between individuals is required, based on the variables selected. Here, a Gower distance matrix was used^49^, due to its utility with mixed categorical and continuous data. Gower distance is calculated as an average of the distances between two individuals calculated for each variable being considered. If the variable is continuous, a standardised difference is used (absolute difference divided by the range), and if the variable is categorical, the distance is 1 if the individuals differ, or 0 if they are the same. One drawback of the Gower distance metric is that it is sensitive to outliers and non-normality of continuous variables. Consequentlly, due to the significant right-skewness of substructure weight, the data from this variable were log-transformed to approximate normality; a *log*(*x* *+ 1)* transformation was used due to the presence of zeroes in the data (e.g. from the floating structures).

The PAM algorithm applies the following steps, based on the Gower distance matrix, to assign a population of *n* individuals to *k* clusters:

1. Assign *k* randomly selected individuals as cluster medoids.
2. Assign all remaining *n-k* individuals to the cluster with the most proximate medoid.
3. Reassign as medoid the individual in each cluster which would yield the lowest average distance for that cluster.
4. If a change is made at step 3, return to step 2.

In order to select the optimum number of clusters, the average silhouette width of the population was calculated when arranged into 2–25 clusters. Silhouette width is a measure of the closeness of each individual to the rest of the individuals in its cluster, relative to their closeness to the individuals of the nearest neighbouring cluster. It is calculated as:

$$s\left(i\right)= \frac{b\left(i\right)-a(i)}{b(i)}$$

where for individual *i*, *s(i)* is the silhouette width, *a(i)* is the average dissimilarity from other members of *i*’s assigned cluster, and *b(i)* is the average dissimilarity from the members of the nearest neighbouring cluster, i.e. the minimum average dissimilarity between *i* and the members of each of the other clusters to which *i* was not assigned. The algorithm was applied using the ‘cluster’ package in the R statistical programming language^50^.

## Results and discussion

Examining the average silhouette width revealed 13 to be the optimum number of clusters. These clusters, as assigned using the PAM algorithm, can be characterised using their medoids as an exemplar individual from the group (Table 1), similar in interpretation to the median of the group.

**Table 1:** Characteristics of the medoids of 13 clusters classifying the 552 oil and gas structures in the North Sea.

Cluster	Category	Status	Product	Substructure weight (t)	N
1	Fixed steel	Operational	Oil	5180	144
2	Fixed steel	Closed down	Oil	3225	18
3	Fixed steel	Decommissioned	Oil	6155	8
4	Fixed steel	Operational	Gas	1050	215
5	Fixed steel	Closed down	Gas	665	34
6	Fixed steel	Decommissioned	Gas	5094	13
7	Fixed steel	Operational	Condensate	5000	26
8	Floating steel	Operational	Oil	14,000	12
9	Floating steel	Operational	Oil	0	29
10	Floating steel	Decommissioned	Oil	0	19
11	Floating steel	Operational	Gas	7500	7
12	Gravity-based concrete	Operational	Oil	254,000	19
13	Gravity-based concrete	Operational	Gas	241,360	8

Note, these are akin to the median of the cluster—not all individuals in a cluster will share identical values across all variables.

**Figure 1:**
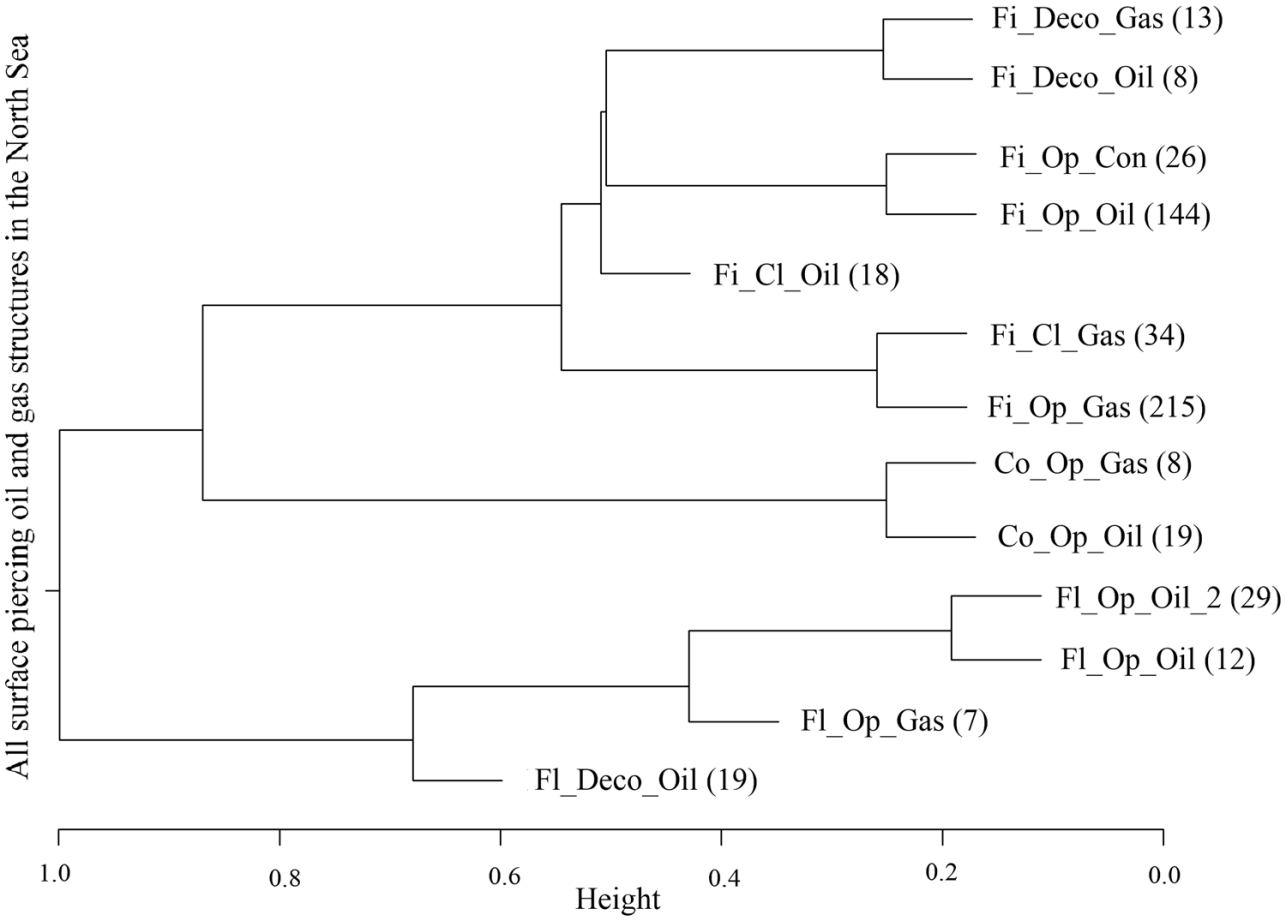
Dendrogram displaying the distances between medoids of the clusters, as arranged by the clustering algorithm. The properties of the medoids are designated as structure type_status_product, with the abbreviations being used: Fi, Fl and Co for Fixed steel, Floating steel and Concrete gravity base; Op, Cl and Deco for Operational, Closed down and Decommissioned; and Oil, Gas and Con being Oil, Gas, and Condensate. The number of structures in the cluster (n) is given in brackets.

**Figure 2:**
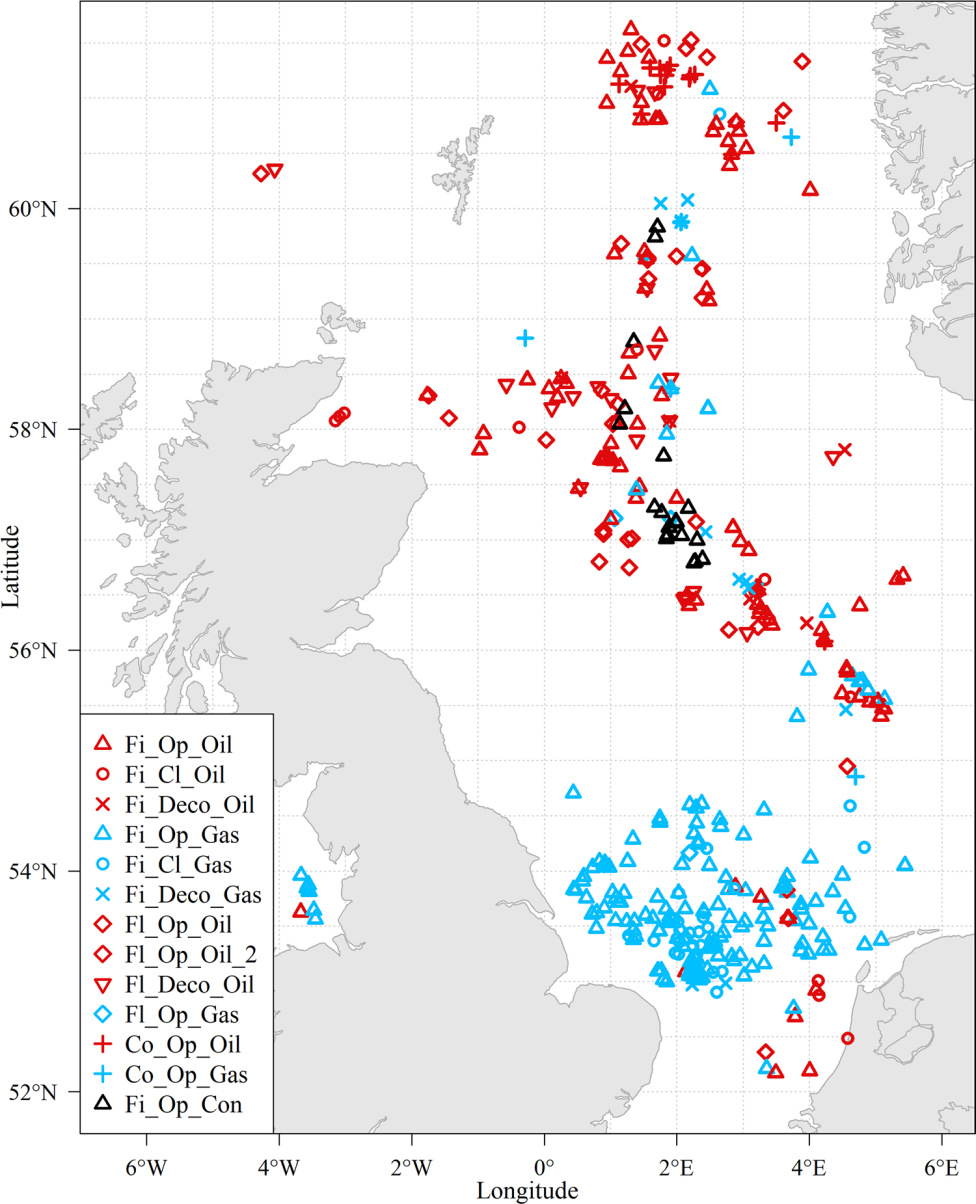
Map of oil and gas platforms in the North Sea. Symbols denote the cluster to which the platform was assigned during the clustering process, and are coloured by hydrocarbon product. The clusters are designated as structure type_status_product, with the abbreviations being used: Fi, Fl and Co for Fixed steel, Floating steel and Concrete gravity base; Op, Cl and Deco for Operational, Closed down and Decommissioned; and Oil, Gas and Con being Oil, Gas, and Condensate. The map was generated using the ‘maps’ and ‘mapdata’ packages in R (v4.1.2; https://www.r-project.org).

Using complete-linkage clustering, it is possible to build a dendrogram using the separation between the medoids hierarchically based on their Gower distances, to show the how clusters relate to one another in distance (Fig. 1). The most important variable for differentiating clusters was structure type (floating or fixed steel, concrete); the two largest splits separate out first the floating platforms, then the concrete platforms. Examining the spatial distribution of the various clusters, the most obvious spatial trend is a north–south split of oil and gas respectively (Fig. 2).

A formal typology of the oil and gas platforms of the North Sea was created, classifying the 552 individual platforms into 13 clusters. With this typology, and the relative numbers of platforms in each cluster (Table 1), it is possible to select a representative subsample of structures as part of the survey design process for a study which is unable to visit the entire population of platforms. Alternatively, if a subsample has already been selected or sampled, or a survey designer does not have complete freedom to choose which platforms can be surveyed, the representativeness of a sample can be assessed, and so the applicability of the results to the wider population can be highlighted.

The variables selected here were relatively basic, dealing only with some aspects of the platforms’ physical size and structure, as well as the hydrocarbon product. For each specific application, a set of variables which are likely to be important in the context of the ecological question being asked should be selected, where available. One difficulty in this, is that the currently available publicly accessible databases (e.g. the OSPAR and OGA databases) are lacking information on some important variables, are incomplete in their records of others, and indeed are inaccurate in yet others.

For example, for a study of fish around oil and gas platforms, there are factors relating to substances discharged from the platform which may affect the fish populations below them. These included whether the structures are normally manned or unmanned (and so have discharge of organic matter in the form of kitchen waste and black- and grey-water), and whether the platform is permitted to discharge produced water (formation water extracted along with the hydrocarbon product and process chemicals) or reinject it back into the reservoir. These data, however, are not included in any public database, and so would require a significant data-mining effort to collect for the entire population, something which was beyond the scope of this study. It may be possible to gather data on these variables for a small selection of platforms (e.g. by contacting the operators directly) and so they could at least be reported as a factor which may affect the ecology of the platforms, even if their comparability with the wider population of unsampled platforms is unknown.

There are also transient variables which can differ temporally at any given platform but may impact the surrounding environment, particularly mobile species which can vary their distribution over short timescales (whereas sessile organisms cannot). For example, activities such as drilling will emit noise and vibration into the surrounding water, but only whilst they are actively occurring. These activities can vary over a range of timescales, but can extend up to several months at a time of activity or inactivity. While these variables might be impossible to include in a typology (due to both their highly transient nature and the amount of data gathering required for their inclusion, as mentioned above), it is essential that they be considered as important contextual information which may bias the data collected at any given time and location.

Some variables have been deliberately omitted following consideration of their relative importance to the ‘definition’ of each cluster. Water depth, for example, could have been included due to the potential influence it would have on the ecology of the system around a platform^51–56^. Additionally, platform location (latitude, longitude, or both) could have been included in the clustering process, as they will affect the ecology of the site^57,58^. These variables were omitted, however, because they are more descriptors of the environment itself, than of the platform. It was decided therefore, that only information about the platform itself would be used for clustering, and the environmental variables can be controlled for (or investigated) as part of the survey design or data analysis of the environmental study. For example, it will be important to look at the distribution of water depths in each cluster, post-hoc, and ensure that representative samples (in particular in the event of a bimodal distribution in the depth data of a given cluster) are selected.

One thing that became apparent over the course of this study is the need for high quality, accurate, publicly accessible databases to be maintained, so that the sort of analysis carried out here can be conducted for future studies using case-appropriate variables for clustering. Much of the information resulting from ecological studies of oil and gas infrastructure may be limited by the number of platforms sampled and a lack of clarity over the respresentativeness of the subsample selected. The current readily accessible databases, while a useful starting point, are limited in the number of potentially ecologically relevant variables they contain, and there are some issues with the accuracy and maintenance of some of the datasets contained therein (e.g. the location data in the OSPAR inventory of the offshore installations contains numerous inaccuracies).

## Conclusions

A typology of oil and gas structures in a given study area (here, the North Sea) is essential for selecting a subsample which is suitably representative of the wider ‘population’. This will increase the extent to which the conclusions drawn from a study can be generalised, allowing the more efficient use of limited resources available for such studies. The work highlights the need for high quality, accurate databases of information about offshore oil and gas infrastructure to be maintained (including a range of relevant variables) so that a similar typology can be created using any and all characteristics deemed of importance to a new study.

## Data Availability

The data analysed in this article are freely available online from the OSPAR Data and Information Management System (https://odims.ospar.org/en/).

## References

[1] Heery EC (2017). Identifying the consequences of ocean sprawl for sedimentary habitats. J. Exp. Mar. Biol. Ecol..

[2] Bulleri F, Chapman MG (2010). The introduction of coastal infrastructure as a driver of change in marine environments. J. Appl. Ecol..

[3] Angus NM, Moore RL (1982). Scour repair methods in the Southern North Sea. Proc. Annu. Offshore Technol. Conf..

[4] Andersson MH, Berggren M, Wilhelmsson D, Öhman MC (2009). Epibenthic colonization of concrete and steel pilings in a cold-temperate embayment: A field experiment. Helgoland Mar. Res..

[5] Andersson MH, Öhman MC (2010). Fish and sessile assemblages associated with wind-turbine constructions in the Baltic Sea. Mar. Freshw. Res..

[6] Connell SD (2001). Urban structures as marine habitats: An experimental comparison of the composition and abundance of subtidal epibiota among pilings, pontoons and rocky reefs. Mar. Environ. Res..

[7] McDougall KD (1943). Sessile Marine invertebrates of Beaufort, North Carolina: A study of settlement, growth, and seasonal fluctuations among pile-dwelling organisms. Ecol. Monogr..

[8] Petersen JK, Maim T (2006). Offshore windmill farms: Threats to or possibilities for the marine environment. Ambio.

[9] Sedano F, Navarro-Barranco C, Guerra-García JM, Espinosa F (2020). From sessile to vagile: Understanding the importance of epifauna to assess the environmental impacts of coastal defence structures. Estuar. Coast. Shelf Sci..

[10] Pastor J, Koeck B, Astruch P, Lenfant P (2013). Coastal man-made habitats: Potential nurseries for an exploited fish species, *Diplodus sargus* (Linnaeus, 1758). Fish. Res..

[11] Bouchoucha M, Darnaude AM, Gudefin A, Neveu R, Verdoit-Jarraya M, Boissery P, Lenfant P (2016). Potential use of marinas as nursery grounds by rocky fishes: Insights from four *Diplodus* species in the Mediterranean. Mar. Ecol. Progr. Ser..

[12] Guidetti P, Bussotti S, Boero F (2005). Evaluating the effects of protection on fish predators and sea urchins in shallow artificial rocky habitats: A case study in the northern Adriatic Sea. Mar. Environ. Res..

[13] Todd VLG, Warley JC, Todd IB (2016). Meals on wheels? A decade of megafaunal visual and acoustic observations from offshore oil & gas rigs and platforms in the North and Irish Seas. PLoS ONE.

[14] Love MS, Schroeder DM, Lenarz WH (2005). Distribution of bocaccio (*Sebastes paucispinis*) and cowcod (*Sebastes levis*) around oil platforms and natural outcrops off California with implications for larval production. Bull. Mar. Sci..

[15] Paxton AB (2020). Artificial habitats host elevated densities of large reef-associated predators. PLoS ONE.

[16] Friedlander AM, Ballesteros E, Fay M, Sala E (2014). Marine communities on oil platforms in Gabon, West Africa: High biodiversity oases in a low biodiversity environment. PLoS ONE.

[17] Page H, Dugan J, Dugan D, Richards J, Hubbard D (1999). Effects of an offshore oil platform on the distribution and abundance of commercially important crab species. Mar. Ecol. Prog. Ser..

[18] Pondella DJ, Zahn LA, Love MS, Siegel D, Bernstein BB (2015). Modeling fish production for southern California’s petroleum platforms. Integr. Environ. Assess. Manag..

[19] Fujii T, Walls A, Horsfield M (2014). Is there a net benefit from offshore structures?. Soc. Pet. Eng..

[20] Reubens JT, Degraer S, Vincx M (2014). The ecology of benthopelagic fishes at offshore wind farms: A synthesis of 4 years of research. Hydrobiologia.

[21] Daigle ST, Fleeger JW, Cowan JH, Pascal P-Y (2013). What is the relative importance of phytoplankton and attached macroalgae and epiphytes to food webs on offshore oil platforms?. Mar. Coast. Fish..

[22] Claisse JT (2014). Oil platforms off California are among the most productive marine fish habitats globally. Proc. Natl. Acad. Sci. USA.

[23] Burt J, Bartholomew A, Bauman A, Saif A, Sale PF (2009). Coral recruitment and early benthic community development on several materials used in the construction of artificial reefs and breakwaters. J. Exp. Mar. Biol. Ecol..

[24] Claisse JT (2015). Impacts from partial removal of decommissioned oil and gas platforms on fish biomass and production on the remaining platform structure and surrounding shell mounds. PLoS ONE.

[25] Krone R, Gutow L, Brey T, Dannheim J, Schröder A (2013). Mobile demersal megafauna at artificial structures in the German Bight: Likely effects of offshore wind farm development. Estuar. Coast. Shelf Sci..

[26] Bugnot AB (2020). Current and projected global extent of marine built structures. Nat. Sustain..

[27] Halpern BS (2008). A global map of human impact on marine ecosystems. Science.

[28] Jones KR (2018). The location and protection status of earth’s diminishing marine wilderness. Curr. Biol..

[29] Ahiaga-Dagbui DD, Love PED, Whyte A, Boateng P (2017). Costing and technological challenges of offshore oil and gas decommissioning in the U.K. North Sea. J. Constr. Eng. Manag..

[30] Bull AS, Love MS (2019). Worldwide oil and gas platform decommissioning: A review of practices and reefing options. Ocean Coast. Manag..

[31] Jørgensen D (2012). OSPAR’s exclusion of rigs-to-reefs in the North Sea. Ocean Coast. Manag..

[32] OSPAR. *“OSPAR decision 98/3 on the disposal of disused offshore installations.”* Ministerial Meeting of the OSPAR Commission*, OSPAR Convention for the Protection of the Marine Environment of the North-East Atlantic* (1998).

[33] Cordes EE (2016). Environmental impacts of the deep-water oil and gas industry: A review to guide management strategies. Front. Environ. Sci..

[34] Fowler AM, Macreadie PI, Jones DOB, Booth DJ (2014). A multi-criteria decision approach to decommissioning of offshore oil and gas infrastructure. Ocean Coast. Manag..

[35] Sommer B (2019). Decommissioning of offshore oil and gas structures: Environmental opportunities and challenges. Sci. Total Environ..

[36] Ekins P, Vanner R, Firebrace J (2006). Decommissioning of offshore oil and gas facilities: A comparative assessment of different scenarios. J. Environ. Manage..

[37] Fowler AM (2018). Environmental benefits of leaving offshore infrastructure in the ocean. Front. Ecol. Environ..

[38] OSPAR. *OSPAR Inventory of Offshore Installations*. (OSPAR, 2017).

[39] Kaufman L, Rousseeuw PJ (1990). Finding Groups in Data.

[40] Lingelbach K (2021). Effects of the COVID-19 pandemic on psychological well-being and mental health based on a German online survey. Front. Public Health.

[41] Punzón A (2010). Spanish otter trawl fisheries in the Cantabrian Sea. ICES J. Mar. Sci..

[42] Ramdani MA, Abdullah S (2021). Application of partitioning around medoids cluster for analysis of stunting in 100 priority regencies in Indonesia. J. Phys..

[43] Miller K, Huettmann F, Norcross B, Lorenz M (2014). Multivariate random forest models of estuarine-associated fish and invertebrate communities. Mar. Ecol. Prog. Ser..

[44] Shokri, E., Razeghi, M., Raeisi Shahraki, H., Jalli, R. & Motealleh, A. The use of cluster analysis by partitioning around medoids (PAM) to examine the heterogeneity of patients with low back pain within subgroups of the treatment based classification system. *J. Biomed. Phys. Eng*. https://jbpe.sums.ac.ir/article_47497.html (2021). 10.31661/jbpe.v0i0.2001-1047 PMC9923237 36818010

[45] Winker H, Kerwath SE, Attwood CG (2013). Comparison of two approaches to standardize catch-per-unit-effort for targeting behaviour in a multispecies hand-line fishery. Fish. Res..

[46] van de Velden M, D’Enza AI, Markos A (2019). Distance-based clustering of mixed data. Wiley Interdiscip. Rev..

[47] Brasch ME, Peña AN, Henderson JH (2021). Image-based cell subpopulation identification through automated cell tracking, principal component analysis, and partitioning around medoids clustering. Med. Biol. Eng. Comput..

[48] Wright PJ, Christensen A, Régnier T, Rindorf A, van Deurs M (2019). Integrating the scale of population processes into fisheries management, as illustrated in the sandeel, *Ammodytes marinus*. ICES J. Mar. Sci..

[49] Gower JC (1971). A general coefficient of similarity and some of its properties. Biometrics.

[50] R Core Team (2020). R: A Language and Environment for Statistical Computing.

[51] Stachowitsch M, Kikinger R, Herler J, Zolda P, Geutebrück E (2002). Offshore oil platforms and fouling communities in the southern Arabian Gulf (Abu Dhabi). Mar. Pollut. Bull..

[52] Ajemian MJ, Wetz JJ, Shipley-Lozano B, Stunz GW (2015). Rapid assessment of fish communities on submerged oil and gas platform reefs using remotely operated vehicles. Fish. Res..

[53] Meyer-Gutbrod EL (2019). Fish densities associated with structural elements of oil and gas platforms in southern California. Bull. Mar. Sci..

[54] Lewbel GS, Howard RL, Gallaway BJ (1987). Zonation of dominant fouling organisms on northern Gulf of Mexico petroleum platforms. Mar. Environ. Res..

[55] Todd VLG, Lavallin EW, Macreadie PI (2018). Quantitative analysis of fish and invertebrate assemblage dynamics in association with a North Sea oil and gas installation complex. Mar. Environ. Res..

[56] Love MS, Nishimoto MM, Snook L, Kui L (2019). An analysis of the sessile, structure-forming invertebrates living on California oil and gas platforms. Bull. Mar. Sci..

[57] Reiss H, Cunze H, König K, Neumann K, Kröncke I (2011). Species distribution modelling of marine benthos: A North Sea case study. Mar. Ecol. Prog. Ser..

[58] Callaway R (2002). Diversity and community structure of epibenthic invertebrates and fish in the North Sea. ICES J. Mar. Sci..

